# Dermis, acellular dermal matrix, and fibroblasts from different layers of pig skin exhibit different profibrotic characteristics: evidence from *in vivo* study

**DOI:** 10.18632/oncotarget.15389

**Published:** 2017-02-16

**Authors:** Yanhai Zuo, Shuliang Lu

**Affiliations:** ^1^ Shanghai Burns Institute, Rui Jin Hospital, Shanghai Jiao Tong University School of Medicine, Shanghai, China

**Keywords:** acellular dermal matrix, dermal fibroblast, fibrosis, pig, wound healing

## Abstract

To explore the profibrotic characteristics of the autografted dermis, acellular dermal matrix, and dermal fibroblasts from superficial/deep layers of pig skin, 93 wounds were established on the dorsa of 7 pigs. 72 wounds autografted with the superficial/deep dermis and acellular dermal matrix served as the superficial/deep dermis and acellular dermal matrix group, respectively, and were sampled at 2, 4, and 8 weeks post-wounding. 21 wounds autografted with/without superficial/deep dermal fibroblasts served as the superficial/deep dermal fibroblast group and the control group, respectively, and were sampled at 2 weeks post-wounding. The hematoxylin and eosin staining showed that the wounded skin thicknesses in the deep dermis group (superficial acellular dermal matrix group) were significantly greater than those in the superficial dermis group (deep acellular dermal matrix group) at each time point, the thickness of the cutting plane in the deep dermal fibroblast group was significantly greater than that in the superficial dermal fibroblast group and the control group. The western blots showed that the α-smooth muscle actin expression in the deep dermis group (superficial acellular dermal matrix group) was significantly greater than that in the superficial dermis group (deep acellular dermal matrix group) at each time point. In summary, the deep dermis and dermal fibroblasts exhibited more profibrotic characteristics than the superficial ones, on the contrary, the deep acellular dermal matrix exhibited less profibrotic characteristics than the superficial one.

## INTRODUCTION

Hypertrophic scarring (HTS) in humans is a serious problem following trauma, burns, or surgery, and often result in uncomfortable and prolonged rehabilitation periods [1]. Even after having been extensively studied at the level of tissues, molecules, and genes, the mechanism of HTS is still not well understood, rendering treatment difficult [2, 3]. Clinically, injuries to the deep dermis often lead to HTS, whereas superficial wounds are likely to heal with little or no scar formation without surgical intervention [4, 5]. It was also reported that HTS occurred after deep dermal wounds in the female red Duroc pig (FRDP). Moreover, the thickness of the healed wounds in FRDP increased with the wound depth [6]. These phenomena inspired us to consider this question: did the dermis from different layers of skin exhibit different profibrotic characteristics? To resolve this issue, we separated the skin of three FRDPs into six layers and autografted the second and fifth layers. It was observed that the skin thickness of the wound models autografted with the fifth layer was significantly greater than that of the second layer at postoperative weeks 2 and 3 [7]. However, in sharp contrast with the long-term process of cutaneous fibrosis, the research period of this study was relatively short. For a better understanding of the fibrotic properties of the dermis from different layers of skin, we believed that longer studies would be needed, so we sought to provide a solution in this study.

It is well known that the dermis mainly consists of extracellular matrix (ECM) and cellular components, the vast majority of which are dermal fibroblasts (DFs). Thus, what are the roles of ECM and DFs in the process of scarring that derive from different layers of the dermis? On the one hand, acellular dermal matrix (ADM) is derived from dermis from which the cellular population (keratinocytes, vascular endothelium, DFs, etc.) has been removed, and it consists primarily of ECM [8–10]. Compared with traditional ADM, FlexHD Pliable (Musculoskeletal Transplant Foundation), a new generation of human ADM, is derived from the deep dermis [11]. Recently, a report by Liu et al [12]. suggested that the use of FlexHD was an independent risk factor for implant loss in implant-based breast reconstruction. Based on these observations, we hypothesized that ADM derived from the deep dermis (termed the deep ADM) exhibited fewer profibrotic characteristics than that from the superficial dermis (termed the superficial ADM). On the other hand, a large number of *in vitro* studies have shown that cells cultured from the deep dermis (termed the deep DF) produced significantly more collagen and exhibited more profibrotic characteristics than those from the superficial dermis (termed the superficial DF) in humans and pigs, yet *in vivo* studies have been rare [7, 13]. Based on these observations, we hypothesized that autologous implantation of the deep DF showed more profibrotic characteristics than that of the superficial DF.

This study was designed to explore the long-term fibrotic properties of the dermis from different layers of skin and to test our hypotheses mentioned above. Because ❶experiments using pigs are less restricted by ethical restrictions than clinical trials, ❷ the similarities of pig and human skin have long been reported [14–16], and the FRDP has been proposed to be a good animal for *in vivo* studies of wound healing, HTS, and DF heterogeneity [6, 7, 17, 18], ❸ skin of rodents, the common laboratory species, is sufficiently different from human skin in histology, anatomy, and immunology, as well, compared with pig skin, rodent skin is more difficult to be separated into several layers using a dermatome [19–21], we designed the following experiment on 7 FRDPs. To the best of our knowledge, this was the first *in vivo* study to explore the profibrotic characteristics of ADM and DF from different layers of pig skin.

## RESULTS

### Macroscopic observations

**Figure 1:**
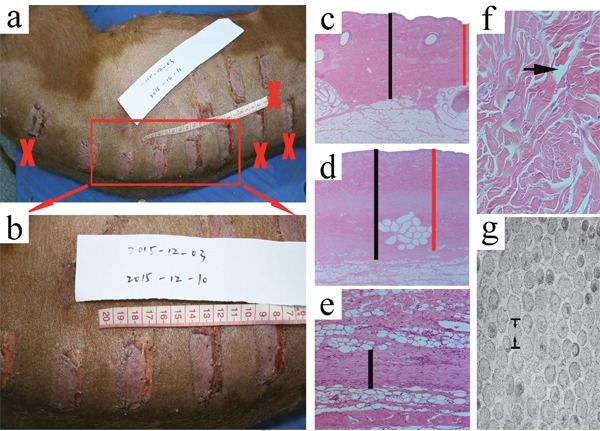
Macroscopic observations of the wound models at the time of 1 week post-wounding and measurement of the primary parameters The macroscopic observations of the wound models at 1 week post-wounding showed that some of the wound models were excluded from the analysis (**a, b**., ×). Following HE staining, the normal skin thickness between the epidermis and the dermal-fat junction and the wounded skin thickness between the epidermis and the remaining fat tissue in the deep/superficial dermis group and in the superficial/deep ADM group were measured under 25 magnification. Both the minimum (**c, d**. red │) and maximum(c, d black │) of the two distances were determined at four randomly selected regions in a blinded fashion. The thickness of the cutting plane in the superficial/deep DF group and the control group was measured in three randomly selected fields in a blinded fashion under 100 magnification (**e**., │). Under 200 magnification, the gap rate between the collagen bundles was measured in three randomly selected fields in a blinded fashion, and was calculated according to the following formula: gap rate = the area of the gap / the area of the whole field x 100% (**f**., arrow). Using TEM, the diameter of the collagen fibrils in either the deep/superficial dermis or the deep/superficial ADM was measured in four randomly selected collagen fibrils under 33000 magnification (**g**., opposite arrow).

All 7 of the FRDPs survived during the study period. A total of 64 (68.82%, 64/93) wound models were included, and 29 wounds were excluded from the analysis (Figure 1a, 1b). A total of 24 samples from the deep/superficial dermis group were harvested at 2 (n=4 and n=4, respectively), 4 (n=5 and n=3, respectively), and 8 (n=4 and n=4, respectively) weeks post-wounding. Twenty-three samples from the deep/superficial ADM group were harvested at 2 (n=5 and n=4, respectively), 4 (n=4 and n=3, respectively), and 8 (n=3 and n=4, respectively) weeks post-wounding. In the deep/superficial DF group and the control group, 17 (80.95%, 17/21) wound models were included (n=7, n=5, and n=5, respectively).

### The thickness of the normal skin, the wounded skin, and the cutting plane

**Figure 2:**
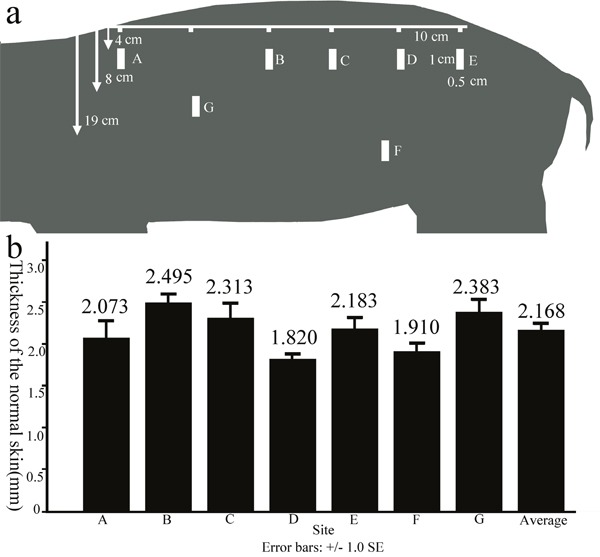
The thickness of the normal skin of FRDPs from different anatomical locations Full-thickness skin samples (1-cm length × 0.5-cm width) were excised from seven zones with distances of 4 cm (site A to site E), 19 cm (site F), and 8 cm (site G) from the median line of the back **a**. The normal skin thicknesses of the FRDPs exhibited dynamic changes according to the anatomical locations, and the mean normal skin thickness of the FRDPs was 2.168±0.320 mm **b**.

Because the normal skin thickness of the FRDPs showed great discrepancy [6, 7], and the normal skin thickness was crucial for the subsequent establishment of the wound models, the thickness of the normal skin from seven anatomical locations (site A to site G) was carefully measured. It was observed that the normal skin thicknesses of the FRDPs were 2.073±0.386 mm(site A), 2.495±0.167 mm(site B), 2.313±0.311 mm(site C), 1.820±0.079 mm(site D), 2.183±0.225 mm(site E), 1.910±0.161 mm(site F), 2.383±0.264 mm(site G), respectively. Taken together, the mean normal skin thickness of the FRDPs was 2.168±0.320 mm (Figure 2).

**Figure 3:**
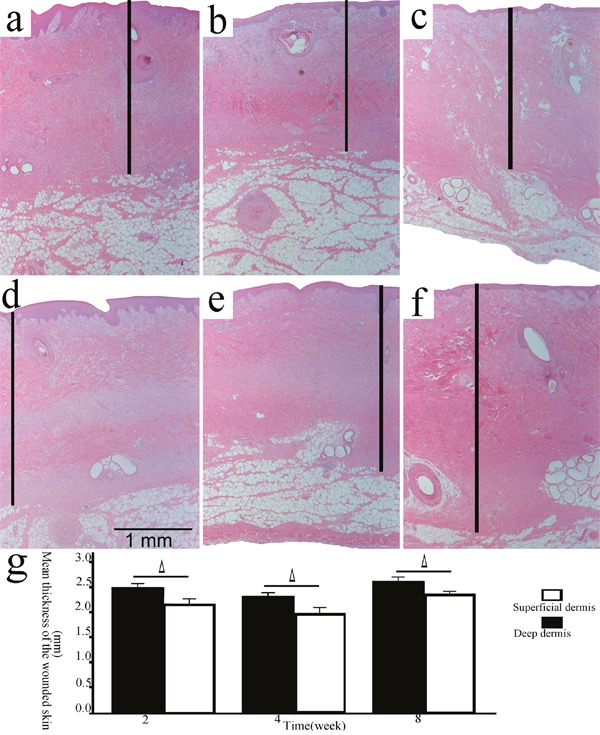
The thickness of the wounded skin samples in the deep/superficial dermis group Representative sections of the wound models in the superficial/deep dermis group at 2, 4, and 8 weeks post-wounding (**a, b, c**., superficial dermis; **d, e, f**., deep dermis; HE, original magnification 25, scale bar: 1 mm, │: the wounded skin thickness). The wounded skin thicknesses in the deep dermis group were significantly greater than those in the superficial dermis group at each time point (**g**., Δ, P<0.05, error bars: +/−1.0 SE).

**Figure 4:**
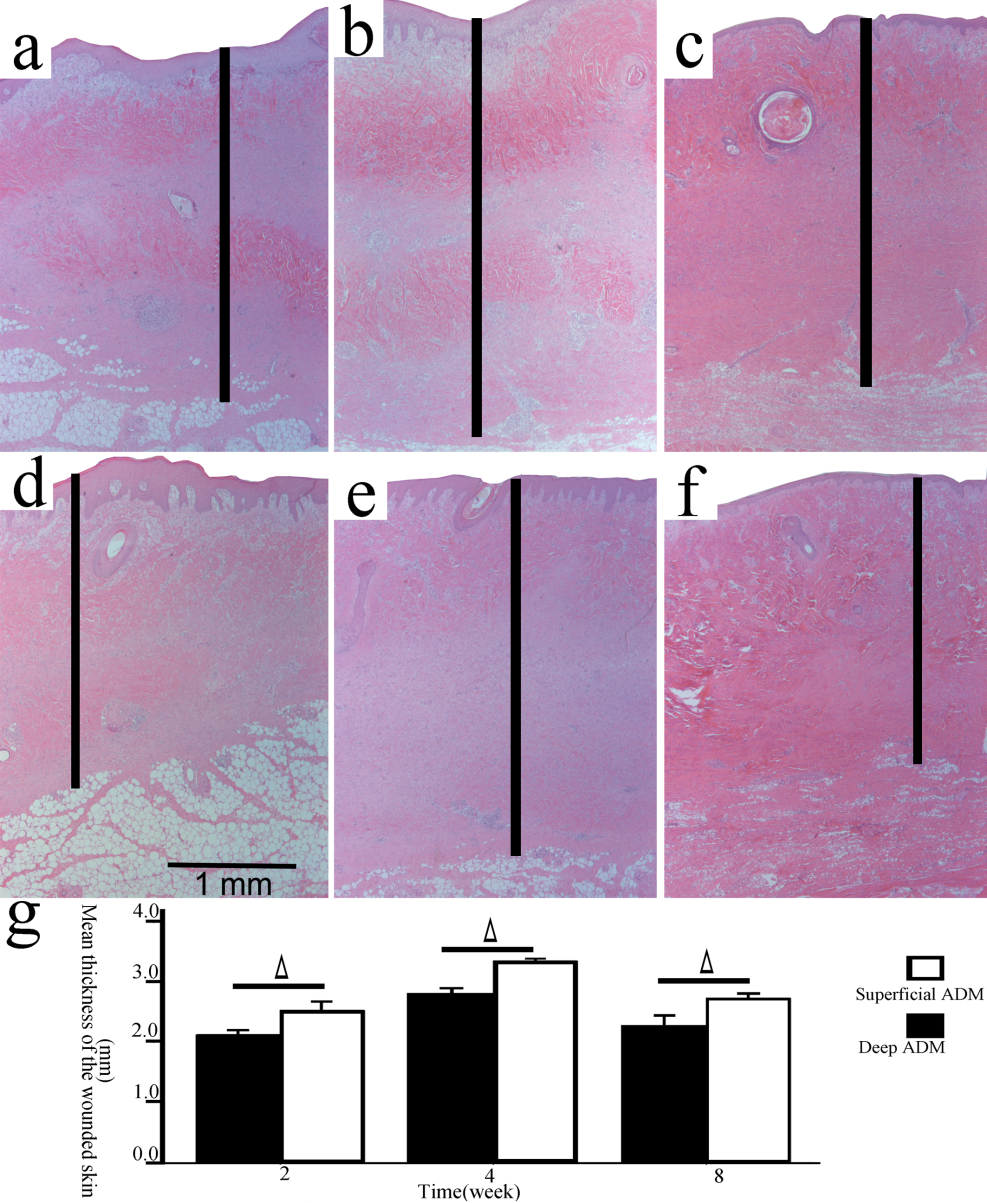
The thickness of the wounded skin samples in the deep/superficial ADM group Representative sections of the wound models in the superficial/deep ADM group at 2, 4, and 8 weeks post-wounding (**a, b, c**., superficial ADM; **d, e, f**., deep ADM; HE, original magnification 25, scale bar: 1 mm, │: the wounded skin thickness). The wounded skin thicknesses in the deep ADM group were significantly less than those in the superficial ADM group at each time point (**g**., Δ, P<0.05, error bars: +/−1.0 SE).

It was reported that the thickness of the healed wounds was an important parameter of cutaneous fibrosis [6, 7], so the wounded skin thickness in the deep/superficial dermis group and the superficial/deep ADM group was measured. It was observed that the wounded skin thicknesses in the deep dermis group were significantly greater than those in the superficial dermis group at 2(2.510±0.138 mm, 2.173±0.219 mm, P=0.040), 4(2.334±0.147 mm, 1.997±0.184 mm, P= 0.028), and 8(2.640±0.156 mm, 2.390±0.080 mm, P= 0.029) weeks post-wounding (Figure 3). Conversely, the wounded skin thicknesses in the deep ADM group were significantly thinner than those in the superficial ADM group at 2(2.126±0.137 mm, 2.528±0.279 mm, P=0.025), 4(2.818±0.147 mm, 3.353±0.049 mm, P= 0.002), and 8(2.280±0.265 mm, 2.735±0.128 mm, P= 0.028) weeks post-wounding (Figure 4).

**Figure 5:**
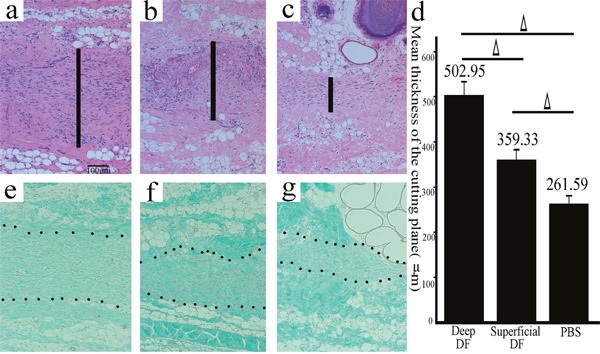
The cutting plane in the deep/superficial DF and the control groups stained by HE and Masson trichrome Representative sections of the wound models in the superficial/deep DF group and the control group (**a**., deep DF group; **b**., superficial DF group; **c**., the control group; HE, original magnification 100, scale bar: 100 μm, │: the thickness of the cutting plane). The thickness of the cutting plane in the deep DF group was significantly greater than that in the superficial DF group and the control group, and the thickness of the cutting plane in the superficial DF group was significantly greater than that in the control group as well (**d**., Δ, P<0.05, error bars: +/−1.0 SE). The cutting plane in each group was stained green by Masson trichrome staining, which strongly suggested that the cutting plane was primarily composed of newly formed collagen (**e**., deep DF group; **f**., superficial DF group; **g**., the control group; Masson trichrome staining, original magnification 100, scale bar: 100 μm).

It was also reported that the extent of fibrogenesis could be determined by the thickness of the cutting plane [22], so the thickness of the cutting plane in the superficial/deep DF group and the control group was measured. It was observed that the thickness of the cutting plane in the deep DF group (502.95±82.31 μm) was significantly greater than that in the superficial DF group (359.33±53.64 μm, P=0.013) and the control group (261.59±43.54 μm, P<0.001), and the thickness of the cutting plane in the superficial DF group was significantly greater than that in the control group (P=0.039) as well (Figure 5a, 5b, 5c, 5d). The cutting plane in each group was stained green by masson trichrome staining, which strongly suggested that the cutting plane was primarily composed of newly formed collagen (Figure 5e, 5f, 5g).

### The location of a-SMA by immunohistochemical staining

**Figure 6:**
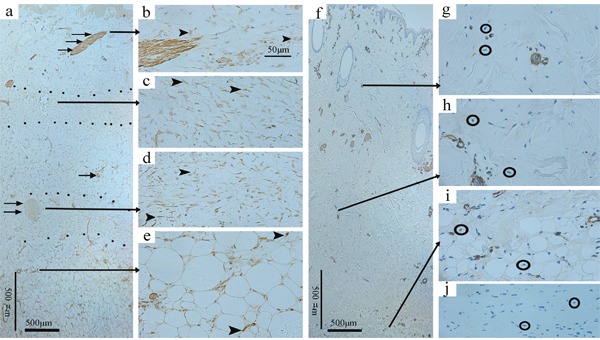
Immunohistochemical staining of a-SMA in the deep dermis group at 2 weeks post-wounding and the normal skin Under 25 magnification, a-SMA mostly localized to cells in the arterioles (single →), the hair follicles (double →), and the arrector pili muscle (triple →), and two cutting planes (dotted areas) could be observed (**a**., original magnification 25, scale bar: 500 μm). Under 200 magnification, widely accumulated a-SMA (arrowheads) was observed in the dermis and adjacent fat tissue (**b-e**., original magnification 200, scale bar: 50 μm), whereas it was absent from the normal skin samples(○) stained with primary antibody (**f**., original magnification 25, scale bar: 500 μm; **g, h, i**., original magnification 200) and the wounded skin samples(○) stained with PBS (**j**., original magnification 200).

Under 25 magnification, a-SMA mostly localized to cells in the hair follicles [23], the arrector pili muscle, and the arterioles. Moreover, two cutting planes could be observed in the deep/superficial dermis and ADM groups at 2 weeks post-wounding (Figure 6a) but none at 8 weeks post-wounding. The cutting plane could also occasionally be observed at 4 weeks post-wounding.

Under 200 magnification, widely accumulated a-SMA was observed in the dermis and the adjacent fat tissue in the deep/superficial dermis and ADM groups at all of the time points (Figure 6b, 6c, 6d, 6e), whereas it was absent from the normal skin samples stained with primary antibody(Figure 6f, 6g, 6h, 6i) and the wounded skin samples stained with buffered saline solution(PBS)(Figure 6j). In particular, the staining intensity of the cutting plane (if present) was obviously higher than that of the adjacent dermis and fat tissue.

### The a-SMA expression by western blotting

**Figure 7:**
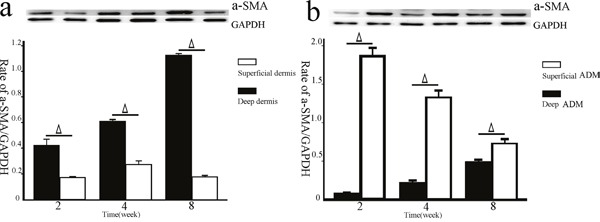
The a-SMA expression in the deep/superficial dermis and ADM groups by western blotting Tissue lysates prepared from the skin samples of the deep/superficial dermis and ADM groups at 2, 4, and 8 weeks post-wounding were subjected to SDS-PAGE and were immunoblotted. Thereafter, all of the lysates were probed with anti-a-SMA monoclonal antibody (dash represents 42 kDa) or anti-GAPDH antibody (dash represents 36 kDa) as a loading control. Quantification analysis showed that the a-SMA expression in the deep dermis group was significantly higher than that in the superficial dermis group at each time point (**a**., Δ, P<0.05, error bars: +/−1.0 SE). Conversely, the a-SMA expression in the deep ADM group was significantly lower than that in the superficial ADM group at each time point (**b**., Δ, P<0.05, error bars: +/−1.0 SE).

Because the error of the data mentioned above might have been increased by subjective bias to some extent, western blotting, a more objective method, was performed in this study. It was observed that the a-SMA expression in the deep dermis group was significantly higher than that in the superficial dermis group at 2 (0.425±0.079, 0.175±0.011, P=0.03), 4(0.611±0.017, 0.273±0.500, P<0.001), and 8(1.126±0.019, 0.179±0.016, P<0.001) weeks post-wounding (Figure 7a). Conversely, the a-SMA expression in the deep ADM group was significantly lower than that in the superficial ADM group at 2(0.087±0.007, 0.219±0.034, P=0.003), 4(0.219±0.034, 1.329±0.146, P=0.004), and 8(0.495±0.021, 0.734±0.071, P=0.005) weeks post-wounding (Figure 7b).

### The histological comparison of dermis and ADM from different layers of pig skin by Hematoxylin & Eosin(HE) staining and electron microscope

**Figure 8:**
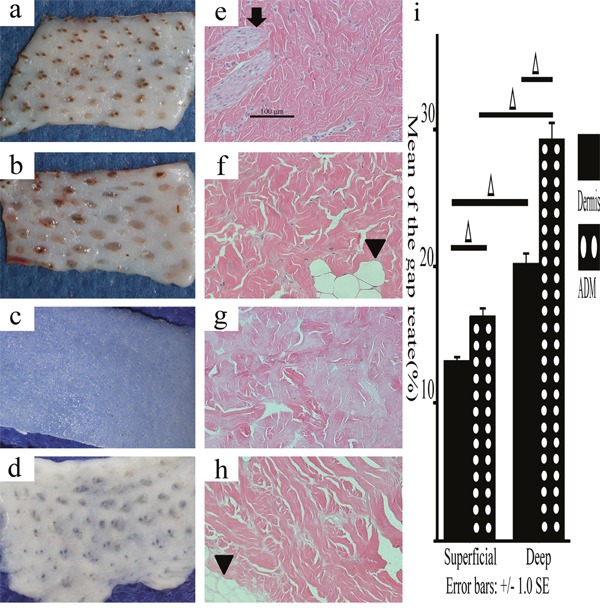
Macroscopic and histological observations of the deep/superficial dermis and ADM Macroscopic observations showed that either the deep dermis or the ADM exhibited more fat domes (arrowheads), which were easy to distinguish from the superficial ones (**a**., superficial dermis; **b**., deep dermis; **c**., superficial ADM; **d**., deep ADM). HE staining showed that the sebaceous gland (↓) and the fat dome (▼) were obvious signs of superficial dermis and deep dermis, respectively. Neither the superficial ADM nor the deep ADM exhibited viable cells or appendage organs, and neither was looser than the superficial/deep dermis (**e**., superficial dermis; **f**., deep dermis; **g**., superficial ADM; **h**., deep ADM, original magnification 200, scale bar: 100 μm). The gap rate in the superficial/deep ADM was significantly greater than that in the superficial/deep dermis. The gap rate in the deep dermis/ADM was significantly greater than that of the superficial layer (**i**., Δ, P<0.05, error bars: +/−1.0 SE).

To investigate the effectiveness of the sodium dodecyl sulfate(SDS) decellularization protocol on either the deep or superficial dermis, to explore the histological comparison of dermis and ADM from different layers of pig skin, and to explain the results above at the level of tissue, both the superficial/deep dermis and the superficial/deep ADM were subjected to HE staining and EM. The sections were carefully examined under 200 magnification, and the results exhibited the absence of viable cells or appendage organs in either the superficial or the deep ADM. In sharp contrast with the dermis, the ADM was a loosened mesh work, consisting of porous collagen fibres. The gap rate of the superficial/deep ADM (16.380±0.879%, 29.287±2.033%) was significantly greater than that in the superficial/deep dermis (13.043±0.477%, 20.160±1.296%)(P=0.036, P=0.019). The gap rate of the deep dermis/ADM was significantly greater than that of the superficial layer (P=0.019, P=0.011) (Figure 8).

**Figure 9:**
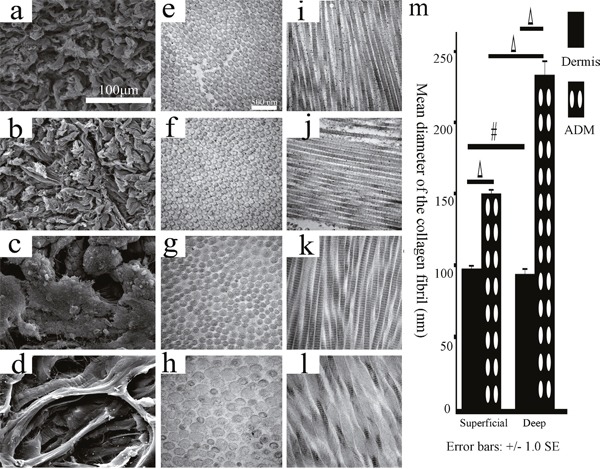
The histological structures of the superficial/deep dermis and ADM of the FRDPs by TEM and SEM The SEM examinations showed that the porous fibres of the superficial and deep dermis exhibited similar disposition (**a**., superficial dermis; **b**., deep dermis; original magnification 1000, scale bar: 100 μm), and the porous fibres of the deep ADM were obviously greater than those of the superficial ADM (**c**., superficial ADM; **d**., deep ADM; original magnification 1000, scale bar: 100 μm). TEM examination showed that the diameter of the collagen fibrils of the superficial/deep ADM was significantly greater than that of the superficial/deep dermis, and the diameter of the collagen fibrils of the deep ADM was significantly greater than that of the superficial ADM (**e, i**., superficial dermis; **f, j**., deep dermis; **g, k**, superficial ADM; **h, l**., deep ADM; original magnification 33000, scale bar: 500 nm, m, #, P>0.05, Δ, P<0.05, error bars: +/−1.0 SE).

The scanning electron microscope(SEM) examinations confirmed the results of the HE staining. Under 1000 magnification, the porous fibres of the superficial and deep dermis exhibited similar disposition, whereas the porous fibres of the deep ADM were obviously greater than those of the superficial ADM (Figure 9a, 9b, 9c, 9d). The transmission electron microscope(TEM) examination showed that the diameter of the collagen fibrils of the superficial/deep ADM(150.44±5.38 nm, 233.5±20.73 nm) was significantly greater than that of the superficial/deep dermis (98.21±3.89 nm, 94.7±7.21 nm)(P=0.001, P<0.001). Although the diameter of the collagen fibrils of the superficial and deep dermis exhibited no significant difference (P=0.928), the diameter of the collagen fibrils of the deep ADM was significantly greater than that of the superficial ADM (P=0.001) (Figure 9e-9m).

## DISCUSSION

In this study, we managed to conduct an *in vivo* study of the profibrotic characteristics of dermis, ADM, and DF from different layers of pig skin. Our data showed that the deep dermis and DFs exhibited more profibrotic characteristics than the superficial ones, on the contrary, the deep ADM exhibited less profibrotic characteristics than the superficial one. Besides, the histological difference between the superficial and deep ADM/dermis was obvious. It was inferred that the histological difference between the superficial and deep ADM/dermis might lead to different profibrotic characteristics of the superficial and deep ADM/dermis.

Zhu, et al. reported that deep wounds of the FRDPs made thick healed wounds, which were thicker than the normal skin and showed similarities to human HTS [6]. However, our data showed that the overall thickness of the healed wound was normal much if any greater than the normal skin. The reason of this inconsistency might be summarized to this: firstly, the epidermis was reserved in our study but completely removed in the former; secondly, the thickness of the discharged dermis in the deep/superficial dermis group(0.75 mm) was less than those in the former(1.1mm or more); thirdly, although the thickness of the discharged dermis in the deep/superficial ADM group(1.5 mm) was more than those in the former, all the wounds were autografted with ADM, which exhibited great potential of inhibiting HTS; last but not the least, the research period of our study(2 month) was shorter than the that of the former(5 month) [6].

### The meaning of the study

The meanings of the study are manifold.

First, to explore the mechanism of HTS, Wang et al. compared the DFs isolated and cultured from the deeper layers of the normal skin with those from HTS, and found that deep DFs of the human skin resembled those of HTS, and they hence suggested that deep DFs might contribute to HTS [13]. Although this study proposed a new perspective for exploring the mechanism of HTS at the level of the cell, it was merely an *in vitro* study, which might weaken the credibility of this new explanation. Our *in vivo* study showed that the deep DFs exhibited greater fibrotic properties than the superficial DFs, which might be a valuable addition to this new explanation.

Second, this study, together with previous studies by Wilson et al [11] and Liu et al [12]., showed the necessity of producing superficial and deep ADM, respectively, and of employing ADM according to clinical need. For example, the superficial ADM might be employed in implant-based treatment of breast reconstruction and atrophic scars. Conversely, the deep ADM might be employed in the treatment of deep wounds prone to healing with HTS.

Third, seeded cells, scaffold materials, and the induction signal are the three basic elements of tissue engineering. Skin tissue engineering is one of the most widely used tissue engineering products, and DFs are among the major seeded cells for skin tissue engineering [24, 25]. Therefore, this study, together with our previous study [7], might facilitate the selection of more suitable scaffold materials and seeded cells for skin tissue engineering.

Fourth, autologous DF-based cellular therapy has been widely employed in the field of repair and regeneration [26]. Thus, this study, together with our previous study [7], might shed light on the selection of more suitable cells according to the therapeutic indication. For example, to treat facial wrinkles, injecting deep autologous DFs might exhibit better therapeutic effects than injecting superficial DFs.

Last but not least, although human DF heterogeneity has long been recognized [27, 28], most of the results were derived from *in vitro* studies, which imposed great restrictions on further studies in this field. In our previous study, we attributed this limitation to the ethical restrictions of clinical trials and the lack of animal models, and we proposed the FRDP as an animal model for further *in vivo* studies of DF heterogeneity [7]. This *in vivo* study showed similar results to the existing *in vitro* studies, which were valuable additions to the existing literature, and we completed studies of DF heterogeneity with a broader perspective available.

### Advantages and disadvantages

The advantages of this study are summarized as follow. First, because allogenic implantation of ADM led to either a short-lived acute inflammatory response or long-term immune responses [29], autologous implantation of dermis, ADM, and DFs was employed in this study, which minimized the errors caused by immunologic factors. Second, all of the data in this study were derived from an *in vivo* study, and it has long been reported that pig skin is sufficiently similar to human skin under both normal and pathological conditions, so the clinical significance of this study was strengthened.

The disadvantages of this study are as follows. First, although the rate of the included wound models in this study (68.82%) was higher than that in our previous study (51.85%) [7], it was still not sufficiently high, mainly because of the difficulties in effectively protecting the wound areas of the pigs in an ethical manner. Second, the study period of the superficial/deep DF group was relatively short, and the final destination of the implanted DFs could not be determined, which might have weakened the credibility of this study. This effect was caused by the difficulties in cultivating so many cells for autologous implantation during this study period, and it will be paid greater attention in our future study. Third, there was potential gender bias. Fourth, the wounds were too close to each other, so it was unclear whether a given wound influenced the healing progress of adjacent wounds or not. Last but not the least, the sample size of this study was relatively small.

## MATERIALS AND METHODS

This study was approved by the Scientific Investigation Board of Shanghai Jiao Tong University School of Medicine, Shanghai, China. 7 FRDPs(20 kg, aged 2 months) were purchased and raised in the laboratory of animal sciences of Shanghai Jiao Tong University School of Medicine on a twelve hours light/dark cycle. The animals had access to a pig grower diet and water freely. As previously described by Zuo et al [7]. and Yuan et al [22]. general anesthesia was initiated through the intramuscular injection of pentobarbital sodium(20 mg/ml, 0.833 ml/kg, Westang Biotechnology Inc., Shanghai, China) and maintained using propofol(4 mg/kg/h, Fresenius Kabi Deutschland GmbH. Germany). Before the surgery, pethidine(1.2 mg/kg, Qinghai Pharmaceutical Co., Ltd., China) was administered via intramuscular injection. During the surgery, the basic vital signs were monitored. To measure the thickness of the normal skin of 3 FRDPs from different anatomical locations, full-thickness skin samples were excised at seven zones at distances of 4 cm (sites A-E), 19 cm (site F), and 8 cm (site G) from the median line of the back(Figure 2) and then were subjected to HE staining.

### Establishment of wound models and sampling

**Figure 10:**
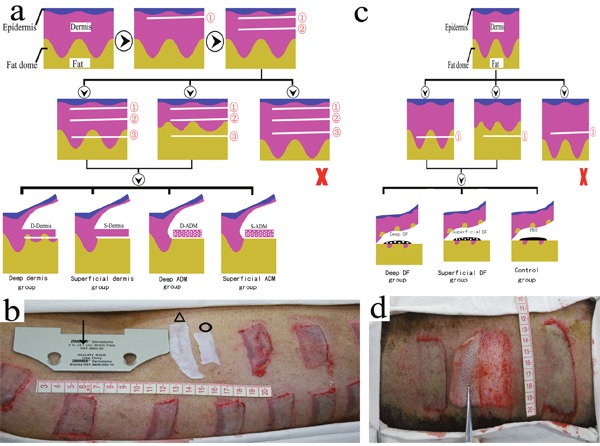
Establishment and grouping of wound models on the FRDPs To explore fibrotic properties of the superficial/deep dermis and ADM, each wound area was horizontally sectioned into three tongue-shaped flaps (2.0 cm×5.1 cm, termed the epidermis-dermis (➀), superficial dermis (➁), and deep dermis (➂), respectively), using a dermatome set at a width of 5.1 cm (↓) and a thickness of 0.75 mm. The wound areas on which the deep dermis did not form a dermis-fat interface were discharged (×). Wound areas with superficial/deep dermis cut from the pedicle were termed the deep/superficial dermis group. Wound areas with both superficial and deep dermis cut from the pedicle and autografted with deep/superficial ADM (Δ, О) were termed the deep/superficial ADM group **a, b**. For the *in vivo* study of the profibrotic characteristics of deep/superficial DFs, each wound area (2.5 cm×6.0 cm) was horizontally sectioned into one skin flap (➀) at the level of the fat layer using a standard roller dermatome, and the flaps that did not form a dermis-fat interface were discharged (×). Wound areas autografted with 5×10^7^/1 ml of superficial/deep DF and 1 ml of PBS were termed the superficial/deep DF and control groups, respectively **c, d**.

To explore the long-term fibrotic properties of the dermis from different layers of pig skin, 36 wound areas (2.0 cm×5.1 cm) were marked on one side of the dorsa of 4 FRDPs. Each wound area was horizontally sectioned into three layers using a dermatome (ZIMMER Surgical. Inc., U.S.A.) set at a width of 5.1 cm and a thickness of 0.75 mm, resulting in three tongue-shaped flaps (2.0 cm×5.1 cm, termed the epidermis-dermis, superficial dermis, and deep dermis, respectively), with pedicles of 5.1 cm in width in the same direction. Wound areas in which the deep dermis did not form a dermis-fat interface were discarded. Wound areas with the superficial/deep dermis cut from the pedicle and discarded were then sutured and termed the deep/superficial dermis group (n=18, n=18, respectively) (Figure 10a, 10b). Full-thickness skin samples were harvested from the centre of the wound areas of both groups at times of 2 (n=6, n=6), 4 (n=6, n=6), and 8 (n=6, n=6) weeks post-wounding, and they were subjected to HE staining, immunohistochemical staining, and western blot. The discharged superficial and deep dermis (Figure 8a, 8b) were subjected to electron microscopy (EM) and HE staining and were used to manufacture ADM.

To explore the fibrotic properties of ADM from different layers of pig skin, both the superficial ADM and deep ADM were made using SDS according to the method previously described [30]. The ADM (Figure 8c, 8d) was subjected to HE staining and EM and was used for the subsequent establishment of wound models. On the other side of the 4 FRDPs, 36 wound areas were created as described above. Thereafter, both the superficial dermis and the deep dermis were simultaneously cut from the pedicle and discharged. The wound areas were then autografted with the superficial/deep ADM and sutured and were termed the superficial/deep ADM group (n=18 and n=18, respectively) (Figure 10a, 10b). Full-thickness skin samples were harvested from the centre of the wound areas of both groups at 2 (n=6, n=6), 4 (n=6, n=6), and 8 (n=6, n=6) weeks post-wounding and were subjected to HE staining, immunohistochemical staining, and western blot.

For the *in vivo* study of the profibrotic characteristics of DF from different layers of pig skin, superficial and deep DFs were cultured from one side of the dorsa of 3 FRDPs as previously described by Zuo et al [7]. Cells from passages 5–8 were used in this study. A total of 21 wound areas (2.5 cm×6.0 cm) were marked on the other dorsa of the 3 FRDPs. Each wound area was horizontally sectioned into one skin flap at the level of the fat layer, using a standard roller dermatome (Medical Instruments Corp. Ltd., Shanghai, China). The flaps that did not form a dermis-fat interface were discharged. Thereafter, as described by Thangapazham et al. [26], the surface of the fat layer was autografted with 2×10^7^/1 ml of superficial/deep DF and then was covered by the skin flap. The wound areas were then sutured and termed the superficial/deep DF group (n=7 and n=7, respectively). The wound areas treated with 1 ml of PBS served as the control group (n=7) (Figure 10c, 10d). Full-thickness skin samples were harvested from the centre of the wound areas of the three groups at 2 weeks post-wounding and were subjected to HE staining and Masson trichrome staining.

Because the thickness of the skin samples was a primary parameter in this study design, all of the wound areas were dressed with sterile gauze without any compression. After surgery, lincomycin (600 mg, Yishun Pharmaceutical Co., Ltd., Hainan, China) and tramadol (50 mg, Grunenthal GmbH, Germany) were used for 3 days through intramuscular injections. All of the FRDPs were sacrificed immediately after the last sampling. To minimize the error of the healed wound thicknesses, the exclusion criteria included the following: wounds in which the deep dermis did not form a dermis-fat interface when established; wounds with swelling, secretion, subcutaneous hydrops or pus at time of 1 week post-wounding.

### HE staining

All of the skin samples were fixed in 4% neutral buffered formalin for at least 24 hours and then were dehydrated and embedded in paraffin. Vertical sections of 4 μm were subjected to standard HE staining. The images were viewed under 25, 100, or 200 magnification using a Zeiss microscope (Carl Zeiss, Inc., Germany) and were captured with a digital still camera (Nikon Instruments Inc., NY, U.S.A.). Two thicknesses, i.e., the normal skin thickness of the dorsum of the FRDP between the epidermis and the dermal-fat junction (Figure 1c) and the wounded skin thickness between the epidermis and the remaining fat tissue in the deep/superficial dermis group and the superficial/deep ADM group, were measured under 25 magnification (Figure 1d). As the dermis-fat interface exhibited certain variations along a section, both the minimum (Figure 1c, 1d red │) and maximum(Figure 1c, 1d black │) of the two distances were determined at four randomly selected regions in a blinded fashion. To determine the content of the newly formed ECM, the thickness of the cutting plane in the superficial/deep DF group and the control group was measured in three randomly selected fields in a blinded fashion under 100 magnification (Figure 1e). To reflect the tightness of the collagen arrangement, under 200 magnification, the gap rate between the collagen bundles was measured in three randomly selected fields in a blinded fashion, and was calculated according to the following formula: gap rate = the area of the gap / the area of the whole field x 100% (Figure 1f). All of the parameters were determined using ImageJ software (http://rsbweb.nih.gov/ij/download.html) with three repetitions.

### EM

The superficial/deep dermis and ADM were subjected to SEM and TEM. As previously described by Zuo et al. [7], following fixation in 1% (w/v) osmium tetroxide in PBS for 2 hours, the samples were dehydrated in increasing concentrations of ethanol for 10 min at each concentration. For TEM, the observations were conducted using a Philips CM-120 TEM (Philips, Eindhoven, the Netherlands). For SEM, the observations were conducted using a Philips QUANTA-200 SEM (Philips, Eindhoven, the Netherlands). The diameter of the collagen fibrils was measured from four randomly selected collagen fibrils under 33000 magnification (Figure 1g) using ImageJ software with three repetitions.

### Masson trichrome staining

Masson trichrome, a simple histological stain, was primarily used to distinguish collagen fibres and to detect collagen synthesis [31, 32]. To determine whether the cutting plane in the superficial/deep DF and control groups was newly formed collagen deposition, masson trichrome staining was performed as previously described with some modifications [31]. Under 100 magnification, the cutting plane in each group was observed using a Zeiss microscope (Carl Zeiss, Inc., Germany) and was captured with a digital still camera (Nikon Instruments Inc., NY, U.S.A.).

### Immunohistochemical staining

For the location of alpha smooth muscle actin (a-SMA), one of the key profibrotic markers that stimulate the development of fibrotic conditions [33–37], immunohistochemical staining was performed as previously described with some modifications [23, 38]. Briefly, sections (4 μm) were deparaffinized with xylene and rehydrated and incubated with 3% H_2_O_2_ for 20 min to eliminate endogenous peroxidase activity. The sections were then incubated overnight at 4°C with a 1:1500 dilution of primary rabbit anti-a-SMA antibody (ab5694; Abcam, Cambridge, MA, U.S.A.), which was detected with a GTVision TM Ш Detection System/Mo&Rb (GK500705, Gene Tech Co., Ltd., Shanghai, China), as described by the manufacturer's instructions. The normal skin sample stained with primary antibody and the wounded skin sample stained with PBS served as controls. At 25, and 200 magnification, the location of a-SMA in each group was carefully examined using a Zeiss microscope (Carl Zeiss, Inc., Germany) and was captured with a digital still camera (Nikon Instruments Inc., NY, U.S.A.).

### Western blotting

For quantitative analysis of a-SMA, equal amounts of solubilized protein from each sample were loaded onto a 10% SDS-PAGE gel (PG 112, Yichen Biotech Co., Ltd., Shanghai, China) and then were transferred to a polyvinylidene difluoride membrane (ISEQ00010, Merck Millipore Ltd., Darmstadt, Germany). The membrane was then incubated with rabbit anti-a-SMA (1:1000, ab5694; Abcam, Cambridge, MA, U.S.A.), HRP-conjugated GAPDH rabbit mAb (1:1000, 3638S; Cell Signaling Technology, Inc., MA, U.S.A.), and donkey anti-rabbit IgG H&L (HRP) antibody (1:1000, ab6802; Abcam, Cambridge, MA, U.S.A.). Thereafter, the bands were probed using ECL Western Blotting Substrate (32109, Thermo Fisher Scientific, MA, U.S.A.) and were visualized by exposing the membranes to a Tanon 5500 Chemiluminescent Imaging System (Tanon Science & Technology Co., Ltd., Shanghai, China). The blots were scanned, and the band intensity was quantified using ImageJ software. The a-SMA expression in skin samples was calculated according to the following formula: a-SMA expression=the band intensity of a-SMA/the band intensity of GAPDH.

### Statistics

All data were presented as means ± standard deviations and processed using SPSS 13.0 for Windows(SPSS, Chicago, IL, U.S.A). For the analyses of the wounded skin thicknesses, and the a-SMA expression in the deep/superficial dermis group, and the superficial/deep ADM group, the independent sample t-test was employed. For the analyses of the thickness of the cutting plane in the superficial/deep DF group, and the control group, and the analyses of the diameter of the collagen fibril and the gap rate in the deep/superficial dermis, and the superficial/deep ADM, the one-way analysis of variance and Dunnett's multiple comparison test was employed. A two-tailed P-value <0.05 was considered significant.

## Abbreviations

ADM: acellular dermal matrix
α-SMA: α-smooth muscle actin
DF: dermal fibroblasts
ECM: extracellular matrix
EM: electron microscopy
FRDP: female, red Duroc pig
H&E: Hematoxylin & Eosin
HTS: Hypertrophic scarring
PBS: phosphate buffer saline solution
SDS: sodium dodecyl sulfate
SEM: scanning electron microscope
TEM: transmission electron microscope.
